# The impact of minimally-invasive esophagectomy operative duration on post-operative outcomes

**DOI:** 10.3389/fsurg.2024.1348942

**Published:** 2024-02-19

**Authors:** Haley I. Tupper, Belia O. Roybal, Riley W. Jackson, Kian C. Banks, Hyunjee V. Kwak, Nathan J. Alcasid, Julia Wei, Diana S. Hsu, Jeffrey B. Velotta

**Affiliations:** ^1^ Division of General Surgery, Department of Surgery, University of California, Los Angeles, CA, United States; ^2^ Division of Thoracic Surgery, Department of Surgery, Kaiser Permanente Northern California, Oakland, CA, United States; ^3^ Division of Research, Biostatistical Consulting Unit, Kaiser Permanente Northern California, Oakland, CA, United States; ^4^ UCSF School of Medicine, University of California, San Francisco, CA, United States; ^5^ Division of General Surgery, Department of Surgery, University of California, San Francisco-East Bay, Oakland, CA, United States; ^6^ Division of Clinical Medicine, Kaiser Permanente Bernard J. Tyson School of Medicine, Pasadena, CA, United States

**Keywords:** minimally-invasive esophagectomy, esophageal cancer, operative duration, post-operative outcomes, anastomotic leak, respiratory infection, mortality

## Abstract

**Background:**

Esophagectomy, an esophageal cancer treatment mainstay, is a highly morbid procedure. Prolonged operative time, only partially predetermined by case complexity, may be uniquely harmful to minimally-invasive esophagectomy (MIE) patients for numerous reasons, including anastomotic leak, tenuous conduit perfusion and protracted single-lung ventilation, but the impact is unknown. This multi-center retrospective cohort study sought to characterize the relationship between MIE operative time and post-operative outcomes.

**Methods:**

We abstracted multi-center data on esophageal cancer patients who underwent MIE from 2010 to 2021. Predictor variables included age, sex, comorbidities, body mass index, prior cardiothoracic surgery, stage, and neoadjuvant therapy. Outcomes included complications, readmissions, and mortality. Association analysis evaluated the relationship between predictor variables and operative time. Multivariate logistic regression characterized the influence of potential predictor variables and operative time on post-operative outcomes. Subgroup analysis evaluated the association between MIE >4 h vs. ≤4 h and complications, readmissions and survival.

**Results:**

For the 297 esophageal cancer patients who underwent MIE between 2010 and 2021, the median operative duration was 4.8 h [IQR: 3.7–6.3]. For patients with anastomotic leak (5.1%) and 1-year mortality, operative duration was elevated above the median at 6.3 h [IQR: 4.8–8.6], *p* = 0.008) and 5.3 h [IQR: 4.4–6.8], *p* = 0.04), respectively. In multivariate logistic regression, each additional hour of operative time increased the odds of anastomotic leak and 1-year mortality by 39% and 19%, respectively.

**Conclusions:**

Esophageal cancer is a poor prognosis disease, even with optimal treatment. Operative efficiency, a modifiable surgical variable, may be an important target to improve MIE patient outcomes.

## Introduction

Esophageal cancer is the sixth leading cause of cancer mortality globally and in 2023, 21,560 new cases were diagnosed in the United States (1, 2). Esophagectomy is the standard of care for resectable disease and in 2010, 64% of esophageal cancer patients in the Surveillance, Epidemiology and End-Results (SEER) database underwent esophagectomy (3). The proportion of esophagectomies performed minimally-invasively (MIE) has steadily increased from 26.9% in 2012 to 55.9% in 2015 (4, 5). Esophagectomy is a highly morbid procedure with long average operative times and high complication rates. Prolonged open esophagectomy operative time is associated with many undesirable post-operative complications, including unplanned reoperation, prolonged intubation, increased anastomotic leak, increased pneumonia, and elevated mortality (6, 7). Operative duration is only partially pre-determined by case complexity and machine learning indicates that surgeon factors are influential, potentially modifiable, variables (8). There is a lack of robust evidence in the existing literature about the effect of MIE operative times on post-operative outcomes. Understanding this relationship will become increasingly important as MIE becomes the standard approach for more surgeons (6, 9, 10). The aim of this study was to characterize the relationship between MIE operative (cut-to-close) time and key post-operative outcomes. We hypothesized that shorter MIE operative time would be associated with decreased rates of complications and mortality.

## Methods

This multi-center retrospective cohort study evaluated patients aged 19 and older with esophageal cancer who underwent minimally-invasive esophagectomy between January 1, 2010 and December 31, 2021. Ethical review was performed by the Kaiser Permanente Northern California Region Institutional Review Board (# 1979549-6). Informed consent was waived given impracticability and no more than minimal risk to included patients.

Data was extracted from institutional records, including the Kaiser Permanente Northern California (KPNC) Cancer Registry, and through retrospective chart review by medical professionals. KPNC encompasses 21 discrete hospitals and provides care to 32.5% of Northern California's population (11). Patients 19 and older who had an institutional minimally-invasive esophagectomy operating room procedure code associated with their chart between 2010 and 2021 and a documented diagnosis of esophageal cancer in the KPNC Cancer registry were initially abstracted from KPNC records. Patients who were not KPNC members for 365 days pre-MIE or 90-days post-MIE were excluded, unless their reason for inactive membership was death. Patients were followed until December 31, 2022. The following predictor and outcomes variables were electronically extracted: age, sex, race/ethnicity, body mass index (BMI), Charlson Comorbidity Index Score (CCI), tumor histology, operative approach and technique, operative duration (cut-to-close), post-operative complications and date of death. Structured electronic health record review was then conducted to validate MIE, to collect additional variables, or complete variables with a missing value from electronic abstraction. MIE was defined as any esophagectomy, for which, at minimum, the intrathoracic portion was accomplished thoracoscopically. We included all surgical approaches, specifically Ivor-Lewis (thoracic anastomosis, two-field), McKeown (cervical anastomosis, three-field), and transhiatal (cervical anastomosis, two-field).

In addition to validation and completion of missing variables, the following predictor and outcomes variables were collected by electronic chart review: Prior cardiothoracic surgery, neoadjuvant chemotherapy, neoadjuvant radiation, adjuvant therapy, preoperative clinical Tumor, Node, and Metastasis (TNM) stage, post-operative pathological TNM stage, hospital readmission or emergency department presentation within 30-days post-MIE, and post-operative complications. Post-operative complications included medical complications (acute kidney injury (AKI), peri-operative myocardial infarction (MI), new atrial fibrillation (A. Fib), and respiratory infection) and surgical complications [anastomotic leak, conduit necrosis and surgical site infection (SSI)]. No data was missing after the chart review.

BMI was stratified into <19, 19–30 and >30 based on clinical classifications of underweight, normal/overweight and obese, and CCI was stratified into four ordinal categories: 0 (none), 1–2 (mild), 3–4 (moderate), and 5+ (severe). Operative duration was treated as a continuous variable with stratification into >4 h or ≤4 h in sub-analysis, described in additional detail below. Clinical stages III and IV were collapsed into stage III+ based on frequent clinical T stage ambiguity in the setting of malignant stricture or mass impeding full endoscopic evaluation. Although we report on pathologic stage, we do not consider this to be a baseline predictor variable as it is determined post-operatively and patients are treated pre-operatively according to their clinical stage.

Key predictor variables were patient demographics (age, sex, race/ethnicity), clinical characteristics (BMI, CCI, prior cardiothoracic surgery), cancer characteristics (clinical stage), and treatment characteristics (neoadjuvant chemotherapy, neoadjuvant radiation). Our primary outcome of interest was mortality (30-day, 90-day and 1-year) and our secondary outcomes of interest were 30-day post-operative complications, particularly anastomotic leak and respiratory infection. We also collected data on length of stay, 30-day hospital readmission and 30-day emergency department (ED) admission. All outcomes except length of stay were dichotomous. The analysis and interpretation of certain key outcomes, specifically AKI, peri-op MI, conduit necrosis, SSI, and 30 and 90-day mortality, were limited by low outcome frequency.

SAS Enterprise 9.4 was used to perform statistical analysis. Continuous variables, specifically age, operative duration and length of stay were found to be non-parametric. The following primary analyses were performed: (1) Descriptive analysis of patient demographic, clinical, cancer or treatment characteristics, reporting on frequency or median with interquartile range (IQR), as appropriate; (2) Association analysis between operative duration and key outcomes, reporting on Wilcoxon rank sum test statistic; (3) Correlation analysis between operative duration and key outcomes, reporting on point-biserial correlation coefficients; (4) Multivariate logistic regression, reporting on odds ratios. Candidate predictor variables for multivariate regression included age, sex, race/ethnicity, BMI, CCI, prior CT surgery, clinical stage, receipt of neoadjuvant chemotherapy, receipt of neoadjuvant radiation and operative duration. For regression analysis, low frequency variables were collapsed as follows: race/ethnicity was collapsed into white or non-white, BMI was collapsed into ≤30 or >30, and CCI was collapsed into 0, 1–2, 3+. Predictor variables for multivariate regression model inclusion were selected based on the Schwarz Bayesian criterion using a forward stepwise selection method and only predictor variables that enhanced overall model fit were included in the final multivariate regression models. Multivariate logistic regression was performed for the outcomes of anastomotic leak, respiratory infection, and 1-year mortality. Sub-group association analysis was also performed, reporting on Chi-square statistics or Fisher's exact, as appropriate, with operative duration stratified into >4 h and ≤4 h. Results were stratified at the 4-hour mark based on prior external research indicating that there are increased complications when thoracic surgeries extend beyond 4 h (12, 13). Kaplan–Meier survival curves using the log-rank test were also generated, comparing surgical duration >4 h and ≤4 h. No adjustments were made for the sampling strategy in the analysis. *P*-values of less than 0.05 were considered to be statistically significant unless otherwise stated and reported confidence intervals are 95% confidence intervals (CI).

## Results

Initially, 368 patients were electronically extracted based on MIE-codes and KPNC cancer registry diagnosis; 70 were excluded after chart review because their esophagectomy was open, not minimally-invasive, and 297 MIE patients were ultimately included in our final study cohort and analyzed.

The median age of study participants was 65.7 years and the majority were male (80.5%) and white (72.4%) (Table 1). Almost 30% were obese (BMI >30), over half (52.2%) had important comorbid conditions (CCI 1+), and prior cardiothoracic surgery was rare (4.4%). Nearly two-thirds (66.0%) had clinical stage III+ cancer and the vast majority received both neoadjuvant chemotherapy (89.6%) and radiation (84.5%), which was reflected by pathologic downstaging, where only 31.7% of cases were pathologic stages III or IV.

**Table 1:** Patient demographic & clinical characteristics.

Total	Overall
	*n* = 297
	*n* (%)
Sociodemographic variables	
Age^a^	65.7 [60.2–72.0]
Female	58 (19.5%)
Race/ethnicity	
Asian/pacific islander	26 (8.8%)
Black	12 (4.0%)
Hispanic/latinx	33 (11.1%)
White	215 (72.4%)
Other	11 (3.7%)
Clinical variables	
BMI	
<19	11 (3.7%)
19–30	199 (67.0%)
>30	87 (29.3%)
CCI	
0	142 (47.8%)
1–2	103 (34.7%)
3–4	34 (11.4%)
5+	18 (6.1%)
Prior CT surgery	13 (4.4%)
Cancer variables	
Histology	
Adenocarcinoma	239 (80.4%)
Squamous	51 (17.2%)
Other	7 (2.4%)
Clinical stage	
I	31 (10.4%)
II	70 (23.6%)
III+	196 (66.0%)
Pathologic stage	
0	28 (9.4%)
I	119 (40.1%)
II	56 (18.9%)
III	81 (27.3%)
IV	13 (4.4%)
Treatment variables	
Neoadjuvant therapy	
Chemotherapy	266 (89.6%)
Radiation	251 (84.5%)
Surgical technique	
Ivor Lewis	237 (79.8%)
McKeown	10 (3.4%)
Transhiatal	50 (16.8%)
Surgical approach	
Total MIE	288 (97.0%)
Hybrid MIE (open abdomen)	9 (3.0%)

^a^ Median [IQR].

The median operative duration was 4.8 h [IQR: 3.7–6.3] and the median length of stay was 3.1 days [IQR: 2.1–5.1]. Complications were relatively infrequent but the most common post-operative complications were new-onset atrial fibrillation (10.1%), respiratory infection (5.7%) and anastomotic leak (5.1%) (Table 2). Peri-operative MI, AKI and conduit necrosis were rare. Early mortality was an uncommon outcome, with 5 (1.7%) and 10 (3.4%) deaths at 30 and 90-days, respectively. Despite the predominance of late-stage cancer, 86.2% of patients survived beyond one year post-MIE.

**Table 2:** Key outcomes (complications, readmissions, mortality) & median operative time.

Total	Total	With outcome	Without outcome	
	*n* = 297	Op time in hours	Op time in hours	
	*n* (%)	Median [IQR]	Median [IQR]	*p*
Post-Op complications				
AKI	5 (1.7%)	4.5 [4.2–5.7]	4.9 [3.6–6.3]	0.72
Peri-Op MI	3 (1.0%)	4.6 [3.3–4.8]	5.0 [3.7–6.3]	0.41
New A. Fib	30 (10.1%)	5.5 [4.4–6.4]	4.8 [3.6–6.2]	0.08
Respiratory infection	17 (5.7%)	6.3 [4.3–7.4]	4.8 [3.6–6.2]	0.07
Anastomotic leak	15 (5.1%)	6.3 [4.8–8.6]	4.8 [3.6–6.2]	0.008
Conduit necrosis	7 (2.4%)	6.9 [4.0–8.5]	4.8 [3.6–6.2]	0.15
SSI	8 (2.7%)	5.0 [4.0–8.4]	4.8 [3.6–6.2]	0.36
Readmissions				
ED	53 (17.8%)	4.8 [3.7–6.3]	5.0 [3.6–6.0]	0.88
Hospital inpatient	41 (13.8%)	4.8 [3.6–6.3]	5.2 [3.9–6.1]	0.45
Mortality				
30-Day	5 (1.7%)	4.6 [4.4–5.7]	4.9 [3.6–6.3]	0.94
90-Day	10 (3.4%)	4.5 [3.9–6.1]	5.0 [3.6–6.3]	0.77
1-Year	41 (13.8%)	5.3 [4.4–6.8]	4.8 [3.6–6.2]	0.04

Operative time was associated with the occurrence of post-operative anastomotic leak and 1-year mortality (Table 2). In general, patients who did not experience the negative post-operative outcome had approximately median operative durations, while patients with undesirable outcomes had operative times that were significantly elevated above the median. Anastomotic leak and 1-year mortality were both associated with prolonged median operative times [6.3 h (*p* = 0.008) and 5.3 h (*p* = 0.04), respectively]. Although only approaching statistical significance, operative times were also prolonged for post-operative new-onset atrial fibrillation and respiratory infection [5.5 h (*p* = 0.08) and 6.3 h (*p* = 0.07), respectively]. Conduit necrosis also trended towards significance [6.9 h (*p* = 0.15)]. ED and hospital readmissions were not associated with differences in operative duration. AKI, peri-op MI, SSI and 30-day and 90-day mortality also demonstrated no difference, although these outcomes were low frequency, limiting interpretation. In correlation analysis, each additional hour of operative time was weakly correlated with increased post-operative complications for all studied complications, except peri-operative MI (Supplementary Table S1).

Multivariate logistic regression was performed to adjust for pre-operative patient characteristics and operative duration for anastomotic leak, respiratory infection and 1-year mortality (Table 3). After adjusting for prior cardiothoracic surgery, receipt of neoadjuvant chemotherapy and receipt of neoadjuvant radiation, each additional hour of surgery was associated with a 19% increase in the odds of 1-year mortality [OR: 1.19 (95% CI: 1.01–1.40)]. After adjusting for age, BMI, and neoadjuvant chemotherapy, each additional hour of surgery was associated with a 39% increase in the odds of anastomotic leak [OR: 1.39 (95% CI: 1.10–1.75)]. Only increasing Charlson comorbidity scores increased the odds of post-operative respiratory infection.

**Table 3:** Multivariate regression analysis of key outcomes.

	Key outcomes		
Potential predictors	Anastomotic leak	Respiratory infection	1-year mortality
	Odds ratio [95% CI]	Odds ratio [95% CI]	Odds ratio [95% CI]
Operative duration^a^	1.39 [1.10–1.75]*	1.21 [0.96–1.52]	1.19 [1.01–1.40]*
Age^a^	0.97 [0.92–1.03]	1.01 [0.95–1.08]	–
BMI			
<30	Reference	–	–
> 30	0.46 [0.12–1.82]	–	–
CCI			
0	–	Reference	–
1–2	–	1.12 [0.28–4.50]	–
3+	–	3.80 [1.03–14.07]*	–
Prior CT surgery			
No	–	–	Reference
Yes	–	–	4.49 [1.30–15.44]*
Neoadjuvant chemotherapy			
No	Reference	Reference	Reference
Yes	0.71 [0.14–3.6]	0.63 [0.07–6.08]	1.87 [0.28–12.39]
Neoadjuvant radiation			
No	–	Reference	Reference
Yes	–	0.55 [0.07–4.45]	1.15 [0.26–5.14]

^a^ Continuous variable.

On subgroup analysis with results stratified into operative time >4 h and ≤4 h, 66.3% of patients (*n* = 197) had operative durations >4 h and 33.7% of patients (*n* = 100) had durations ≤4 h. Demographic and cancer characteristics were balanced between the two cohorts, except BMI (*p* = 0.001) and prior cardiothoracic surgery (*p* = 0.04) (Supplementary Table S2). The median length of stay for patients with operative times >4 h was significantly longer [3.9 days (IQR: 3.0–5.9) vs. 2.2 days (IQR: 2.1–3.1), *p* < 0.001] (Table 4). Prolonged surgeries (>4 h) also had increased proportions of post-operative atrial fibrillation and anastomotic leak, but these associations only approached statistical significance. Kaplan–Meier survival curves also suggested a non-statistically significant survival benefit for surgeries <4 h (*p* = 0.14) but comparison is complicated by differential timing of treatment initiation; surgeries performed in ≤4 h were overwhelmingly performed after regionalization in 2014 (Supplementary Figure S1).

**Table 4:** Key outcomes (complications, readmissions, mortality) for operative time ≤4 h and >4 h.

Post-Op Outcomes	≤4 H	>4 H	*p*
	*n* = 100	*n* = 197	
	*n* (%)	*n* (%)	
LOS^a^	2.2 [2.1–3.1]	3.9 [3.0–5.9]	<0.001
Post-Op complications			
AKI	1 (1.0%)	4 (2.0%)	0.67
Peri-Op MI	1 (1.0%)	2 (1.0%)	1.00
New A.Fib	6 (6.0%)	24 (12.2%)	0.09
Respiratory infection	4 (4.0%)	13 (6.6%)	0.44
Anastomotic leak	2 (2.0%)	13 (6.6%)	0.10
Conduit necrosis	2 (2.0%)	5 (2.5%)	1.00
SSI	2 (2.0%)	6 (3.0%)	0.72
Readmissions			
ED	19 (19.0%)	34 (17.3%)	0.71
Hospital inpatient	11 (11.0%)	30 (15.2%)	0.32
Mortality			
30-Day	1 (1.0%)	4 (2.0%)	0.67
90-Day	4 (4.0%)	6 (3.0%)	0.74
1-Year	8 (8.0%)	33 (16.8%)	0.39

^a^ Median [IQR].

## Discussion

Esophagectomy, an esophageal cancer treatment mainstay, is associated with significant morbidity. Approximately 60% (59%–64%) of patients experience post-operative complications and 90-day mortality after esophagectomy ranges from 4.5%–13% (14, 15). As a poor prognosis cancer with a median survival of only 11 months, minimizing complications is paramount; post-operative complications delay further oncologic treatment, increase mortality, and reduce patients' remaining quality of life. Our multi-center study confirmed a slightly decreased 90-day mortality (3.4%, *n* = 10). Notably, our results highlight that prolonged operative time portends increased odds of post-operative complications, particularly anastomotic leak, and 1-year mortality.

Compared to open esophagectomy, MIE is equivocal or superior on most outcome measures: MIE has equivalent oncologic outcomes, anastomotic leak rate and mortality, and is associated with decreased perioperative blood loss, reduced respiratory infections, shorter length of stay and improved 1-year quality of life (14). However, randomized control trials also highlight that MIE has a longer median operative duration than open (326 min vs. 295 min) (16). Our median MIE time (288 min) more closely resembled reported open esophagectomy durations with one-third of cases requiring 240 min or less.

Prolonged operative duration is associated with increased risk of complications with meta-analysis indicating that the odds of complications increase by 21% with each additional hour of operative time (12). The relationship between operative time and complications is bi-directional and prolonged operative time is only partially predetermined by case complexity (7, 17). Surgeon skill, experience, and attention to deliberate, efficient surgical maneuvers all impact operative duration. Extended operative and anesthesia time may be uniquely harmful to esophagectomy patients for numerous reasons, including increased blood loss, tenuous conduit perfusion, and protracted single-lung ventilation (15, 18).

Our multivariate regression results indicated that, even after adjusting for important independent variables, each additional hour of operative time increased the odds of anastomotic leak by 39%. Anastomotic leak occurs in approximately 10% of esophagectomy patients and is associated with increased length of stay, risk of reoperation, anastomotic stricture, and mortality (19, 20). In particular, anastomotic leak has been shown to be associated with a 3-fold increased risk of 90-day mortality (19). Respiratory infection has also been reported to be the principal cause of death post-esophagectomy, causing 54% of deaths in one large study (15). Although anastomotic leak and respiratory infection lead to increased 90-day mortality, our study did not show increased short term mortality with increased operative duration, due to insufficient study numbers and low 90-day mortality (*n* = 10, 3.4%). Our study did, however, demonstrate that each additional hour of MIE increased the odds of 1-year mortality by 19%.

Our data is derived from 10 years of outcomes in a multi-center, integrated institution that broadly serves communities throughout Northern California, whose member demographics are generally representative of the community (11). Since 2014, our MIE approach, from pre-operative nutrition to surgical technique to post-operative care, has been standardized across the health system through a variety of measures, including esophagectomy regionalization, monthly MIE meetings, double-scrubbing opportunities, and a collaborative learning environment. Although our median MIE operative duration is approximately 38 min faster than reported median MIE times, the data is otherwise generalizable to MIE cases across the United States with roughly comparable patient demographics to those in the SEER esophageal cancer database (21).

The main limitation of this study is the low frequency of key outcomes of interest, a common challenge in esophagectomy studies. Although our data repeatedly suggested that increased post-operative complications were associated with increased operative duration in multiple analyses, we lacked sufficient statistical power to perform robust multivariate logistic regression analyses for many individual complications or 90-day mortality, a particularly important indicator of surgical outcomes (22). By evaluating surgeries that were completed minimally-invasively, rather than open, we may have inadvertently selected for less complex cases, although many variables suggest that these are complex patients (4.4% had undergone prior cardiothoracic surgery, 66.0% were clinical stage III, and 89.6% and 84.5% had received neoadjuvant chemotherapy and radiation, respectively). We also did not collect data on some technical factors that may impact complication rates and mortality, including intra-operative blood loss, ischemic preconditioning and anastomotic technique (23, 24), nor did we include data on facility or surgeon MIE volume (25). However, a prior study in our health system demonstrated that regionalization of esophagectomy care to several Centers of Excellence was associated with decreased rates of complication, while surgeon and facility volume were not (26).

Within our data set, further evaluation of stricture, the most common post-operative complication of esophagectomy, occurring in approximately one-quarter of patients, is warranted (15). Anastomotic stricture may be particularly susceptible to prolonged operative time given the hypothesized role of ischemia on stricture formation. Further data collection to achieve sufficient statistical power to effectively model the relationship between operative duration and key outcomes of interest (e.g., 90-day mortality and conduit necrosis) with multivariate logistic regression is warranted. Beyond additional clarification and verification of the relationship between MIE operative time and key outcomes, surgical centers should begin considering potential interventions to address operative duration, such as surgeon and surgical center feedback on comparative operative duration and outcomes, as well as inter and intra-institutional interventions that facilitate continuous learning between efficient or high-volume surgeons and less-efficient or lower-volume surgeons.

## Conclusion

Esophageal cancer is a devastating disease with a short-life expectancy for patients and their families. Beyond risk stratification, it is imperative that we improve post-operative outcomes. Increased open esophagectomy duration has been shown to be associated with negative post-operative outcomes, including complications and mortality, but the impact of MIE duration has been inadequately characterized. Our study suggests that each additional hour of MIE operative duration increases the odds of anastomotic leak and 1-year mortality by 39% and 19%, respectively, and may be associated with other complications as well. Machine learning indicates that surgeon-specific variables play the largest role (43%) in predicting surgical case duration, followed by procedure type and patient factors (8). Given the association of MIE duration with negative operative outcomes, we must consider how to maximize and standardize thoracic surgeons' MIE operative efficiency to improve patient outcomes.

## Data Availability

The datasets presented in this article are not readily available because Data was accessed with a waiver of informed consent. Per KPNC policy, we are unable to share the data. Requests to access the datasets should be directed to belia.o.roybal@kp.org.
