# Supplementary figures and images for: The impact of minimally-invasive esophagectomy operative duration on post-operative outcomes
Source: Front Surg. 2024 Feb 19;11:1348942. doi: 10.3389/fsurg.2024.1348942 (PMC10909993; doi:10.3389/fsurg.2024.1348942)

# Kaplan-Meier MIE Survival Estimates by Surgery Duration

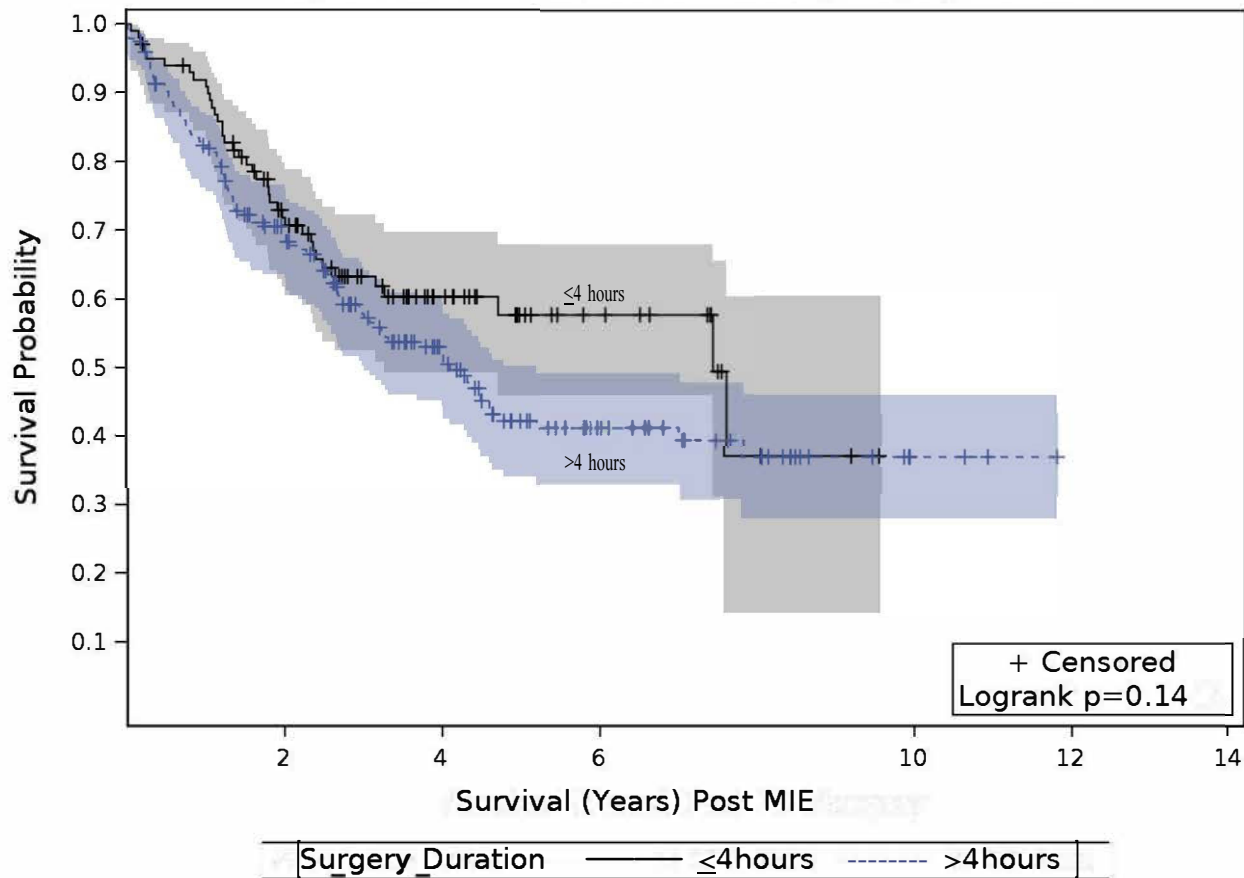

Supplement: Supplementary file 3 [file Image1.pdf]
